# Furfural and 5‐Hydroxymethylfurfural Production from Sugar Mixture Using Deep Eutectic Solvent/MIBK System

**DOI:** 10.1002/open.202100163

**Published:** 2021-10-07

**Authors:** Annu Rusanen, Katja Lappalainen, Johanna Kärkkäinen, Ulla Lassi

**Affiliations:** ^1^ Research Unit of Sustainable Chemistry University of Oulu P.O. Box 4300 FIN-90014 Oulu Finland

**Keywords:** biomass, deep eutectic solvent, furfural, green chemistry, HMF

## Abstract

Choline chloride (ChCl) / glycolic acid (GA) deep eutectic solvent (DES) media with high water content but without any additional catalyst are introduced in furfural and 5‐hydroxymethylfurfural (HMF) production. The effects of water content, reaction time, and reaction temperature are investigated with two feedstocks: a glucose/xylose mixture and birch sawdust. Based on the results, 10 equivalent quantities of water (32.9 wt.%) were revealed to be beneficial for conversions without rupturing the DES structure. The optimal reaction conditions were 160 °C and 10 minutes for the sugar mixture and 170 °C and 10 minutes for birch sawdust in a microwave reactor. High furfural yields were achieved, namely 62 % from the sugar mixture and 37.5 % from birch sawdust. HMF yields were low, but since the characterization of the solid residue of sawdust, after DES treatment, was revealed to contain only cellulose (49 %) and lignin (52 %), the treatment could be potentially utilized in a biorefinery concept where the main products are obtained from the cellulose fraction. Extraction of products into the organic phase (methyl isobutyl ketone, MIBK) during the reaction enabled the recycling of the DES phase, and yields remained high for three runs of recycling.

## Graphical abstract

**Acidic choline chloride/glycolic acid/water deep eutectic solvent** showed promise in furfural production and sawdust fractionation, without any additional catalyst. The achieved furfural yields were high, 62 % from the sugar mixture and 37.5 % from the sawdust. While 5‐Hydroxyfurfural (HMF) yields were low, this is beneficial in the case of sawdust since the treatments appeared to fractionate the sawdust hemicellulose to furfural while most cellulose and lignin remained solid.

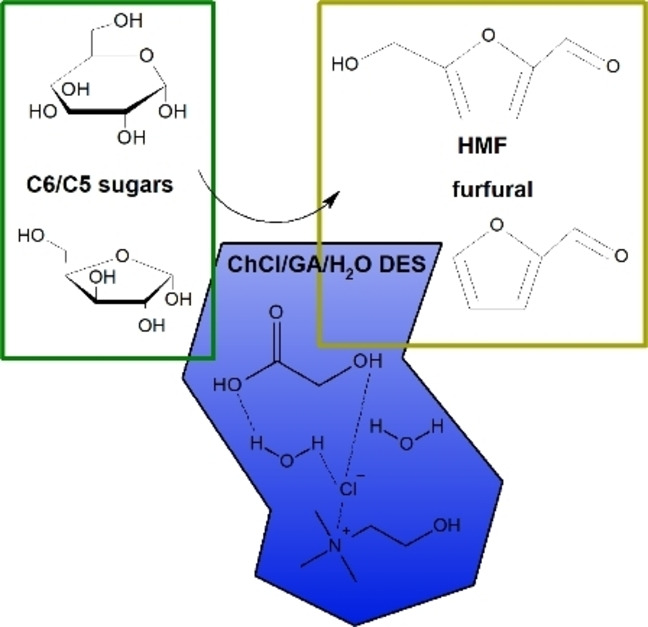

## 1 Introduction

Synthesis of high‐value platform chemicals from lignocellulosic carbohydrates is a major step to achieve more sustainable processes for biorefineries. 5‐Hydroxymethylfurfural (HMF) and furfural are considered key building blocks in biorefineries since they are produced from glucose and xylose, both present in lignocellulosic biomass, and they can be used as a starting material to produce versatile bulk chemicals, solvents, polymers, and fuels.[^1^, ^2^] Current ways to produce HMF and furfural generally involve acid catalysts in aqueous media or solvents such as ionic liquids (ILs).^[3]^ Recently, deep eutectic solvents (DESs) have gathered much interest due to similarly tunable properties as ILs but with cheaper, more abundant, and less hazardous components.^[4]^ DESs, as first proposed by Abbott et al. in 2003, are obtained by mixing together and gently heating two (or more) components in a proper ratio.^[5]^ This straightforward synthesis enables 100 % carbon efficiency, therefore, meeting the principles of green chemistry.^[6]^

DESs are composed of a hydrogen bond acceptor (HBA) and a hydrogen bond donor (HBD) species, and by appropriate selection of these species, it is possible to design DESs of low toxicity and with the desired features. DESs have already been used in biomass processing, for example, in extracting components from biomass and in transformation reactions with eco‐sustainable methods.[^7^, ^8^] Moreover, they are used for fractionation and pretreatment of lignocellulose and in the catalytic conversion of carbohydrates.^[9]^ Usually, an extra catalyst like AlCl_3_, HCl, or H_3_BO_3_ enhances the catalytic conversion of carbohydrates/lignocellulose alongside DES.[^10^, ^11^, ^12^, ^13^, ^14^, ^15^] Fructose has been converted to HMF without an additional catalyst for many years, but recently, xylan was converted to furfural in choline chloride (ChCl)/malic acid (MA) DES with a good yield (75 %).[^16^, ^17^, ^18^] Applying only DES in a conversion reaction relies on the carboxylic acid acting as an HBD, which gives the DES acidic characteristics, and therefore, the DES itself can act as both the solvent and the catalyst.^[18]^ Morais et al. used MA as HBD because of its green credentials and low viscosity compared, for example, to oxalic acid (OA) or citric acid.[^18^, ^19^] However, based on our experiments, ChCl/MA DES requires a higher temperature and much longer time to liquefy and does not stay liquid at room temperature compared to glycolic acid, which was the second best option for furfural production from xylan and stays liquid at room temperature.^[18]^ These features make ChCl/MA DES non‐ideal for industrial use and ChCl/GA a more tempting option to investigate.

One advantage of DES over IL is that DESs are not so water sensitive.^[6]^ Moreover, water is often utilized in DESs since it lowers their viscosity.^[20]^ Water can also enhance certain reactions, and, of course, lowers the price of the solvent. In furfural and HMF production, water has been utilized very varyingly as part of DESs, but primarily with a content from 2.5 to 17 wt.%.[^17^, ^18^, ^21^] Since water is beneficial for hydrolysis and sugar conversion, but an excessive quantity of it also enhances humin formation, an optimal water quantity in DES could enhance the furan compound formation and simultaneously minimize humin formation. However, excess water can rupture the hydrogen bonds between HBD and HBA, so it is essential to verify that DES does not turn to an aqueous solution when a substantial quantity of water is used.[^22^, ^23^]

The aim of this study was to use an environmentally friendly ChCl/GA/H_2_O DES in the conversion of lignocellulosic sugars to HMF and furfural. A high water content (32.9 wt.%) in the DES was used without turning the DES into an aqueous solution. A mixture of glucose and xylose was used to model birch sawdust as a starting material, and experiments with natural birch sawdust were carried out. Generally, HMF yields from biomass or aldoses have been very modest in DES reactions reported in the literature since, contrarily to ketoses, aldoses cannot be directly dehydrated to furanic derivatives but first have to undergo isomerization.^[24]^ Additionally, hexose and pentose sugars (i. e., glucose or xylose) are rarely utilized simultaneously, even if they model the situation in real biomass. The present study aimed to increase the information on simultaneous glucose and xylose conversion to develop the conversion of real biomasses. To the best of our knowledge, ChCl/GA/H_2_O DES has not been utilized either in sugar or in native biomass conversion to furfural or HMF without an additional catalyst.

## 2 Results and Discussion

**Figure 1:**
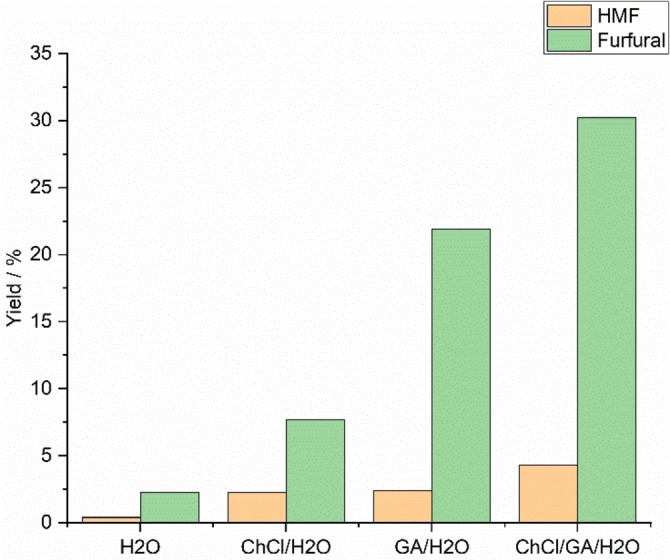
HMF and furfural yields for various aqueous ChCl and GA systems and for a ChCl/GA/H_2_O (1 : 3:0.5) DES.

Control experiments with various aqueous ChCl and GA systems and with a ChCl/GA/H_2_O (1 : 3 : 0.5) DES were performed to demonstrate the influence of acidic DESs on carbohydrate conversion (Figure 1). All control reactions were carried out using a sugar mixture as the feed, the biphasic system with MIBK as the extractive phase, 12.5 mins as the reaction time, and 160 °C as the reaction temperature. Reaction with pure water as the reactive phase gave minor yields (<3%). When ChCl was added to water, both yields increased but were still below 10 %. Glycolic acid in water was a more effective catalyst than ChCl for furfural production (yield increased to 22 %), but the HMF yield did not improve. This result confirmed the hypothesis that the acidic character of reaction media is essential for the dehydration reaction, but that chloride ions can enhance conversion, especially to HMF. When a DES was formed from ChCl, GA, and a small quantity of water, the highest yields were achieved: 30 % for furfural and 4 % for HMF.

### 2.1 Effect of Time and Temperature on HMF and Furfural Formation from Glucose/Xylose Mixture

**Figure 2:**
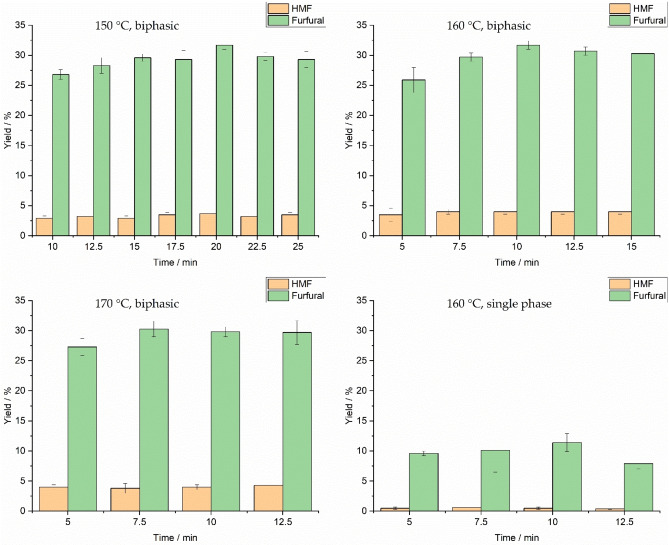
HMF and furfural yields using various conditions such as reaction temperature, time, and phase system (single phase or biphasic).The uncertainty of the results is most likely caused by complicated interactions with ChCl, GA, and water near a regime where DES intermolecular bonding ceases. For example, for the ChCl/urea (1 : 2) DES, discontinuity in the choline–choline and choline–water interactions were observed in a regime between 40–50 wt.% water, while otherwise, the interactions between the DES components weakened and interactions of those with water increased systematically.^[22]^ However, in our study, a quantity of more than 10 equiv. added water started to decrease both yields, which was thus judged as a too high water content. Under these conditions, water perhaps inhibited all interactions between ChCl and GA. Even if different DESs with different components cannot be straightforwardly compared, several studies are consistent with our observations. For example, the ChCl/OA DES was reported to benefit from 16.4 wt.% water addition (which equals our 4 equiv. addition), but after that, the yield of furfural decreased.^[21]^

After the preliminary experiments, a kinetic profile of carbohydrate conversion in the ChCl/GA/H_2_O (1 : 3 : 0.5) DES was determined. A 1 : 3 ratio of ChCl/GA was used in the DES since, presumably, glycolic acid, as a Brønsted acid, would accelerate the dehydration reaction, and therefore, its amount was desired to maximize. A 1 : 2 ChCl/GA eutectic mixture provides the lowest freezing point for the ChCl/GA system, but 1 : 1 and 1 : 3 mixtures are liquid at room temperature and therefore easy to use.^[25]^ Additionally, in a study presented by Morais et al., 1 : 3 ChCl/GA DES (with 5 wt.% H_2_O) was more effective than 1 : 2 ChCl/GA DES (with 5 wt.% H_2_O) for producing furfural from xylan.^[18]^ Reaction temperatures were 150, 160, and 170 °C, and reaction times reported in the literature ranged from 2.5 to 25 mins.[^18^, ^26^] A small quantity of water (0.5 equiv., 2.5 wt.%) was used as part of the DESs because it has been suggested that a small quantity of water weakens the hydrogen bonds between HBD and HBA, making them more available and reactive.^[27]^ Kinetic profiles of HMF and furfural formation are displayed in Figure 2 and based on them, it appears that temperature has quite a limiting effect on HMF and furfural yields. Presumably, all selected temperatures were high enough for enabling conversion, thus not showing significant differences. As expected, the lower temperature of 150 °C required a longer reaction time than the higher reaction temperatures of 160 and 170 °C. However, the effect of time was minor, which is natural when the reaction temperature is high enough. Several experiments were also performed without MIBK as the organic phase, namely, using only DESs as reaction media. In these experiments, furfural and HMF yields were considerably lower compared to the biphasic system. Maximum yields in the single‐phase system were 11 % and 0.5 % for furfural and HMF, respectively, while a similar reaction produced 32 % and 4 % in the biphasic system. Overall, based on these results, the biphasic system, a temperature of 160 °C and a reaction time of 10 min were selected for further reactions.

### 2.2 Effect of DES Water Content on HMF and Furfural Formation from Glucose/Xylose Mixture

Experiments were continued by varying the water content in the DES. The water content of the DES without water addition was found to be 3.2 % due to the water present in ChCl and GA. Therefore, besides the water addition, the DES was also dried in a vacuum and tested in a reaction. Small quantities of added water are common in DESs, since water lowers the viscosity of the solvent.^[20]^ However, the effect of water on DES coordination is unknown for most DESs, and in large quantities, it can cause several reproducibility problems.^[22]^ For the ChCl/urea (1 : 2) DES, the critical quantity is approximately 50 wt.% water, where “water in DES” becomes a “DES in water”.^[22]^ To the best of our knowledge, it has not been determined how far ChCl/GA mixtures can be hydrated before they cease to be DESs on a nanostructural level and become more like aqueous solutions.

**Figure 3:**
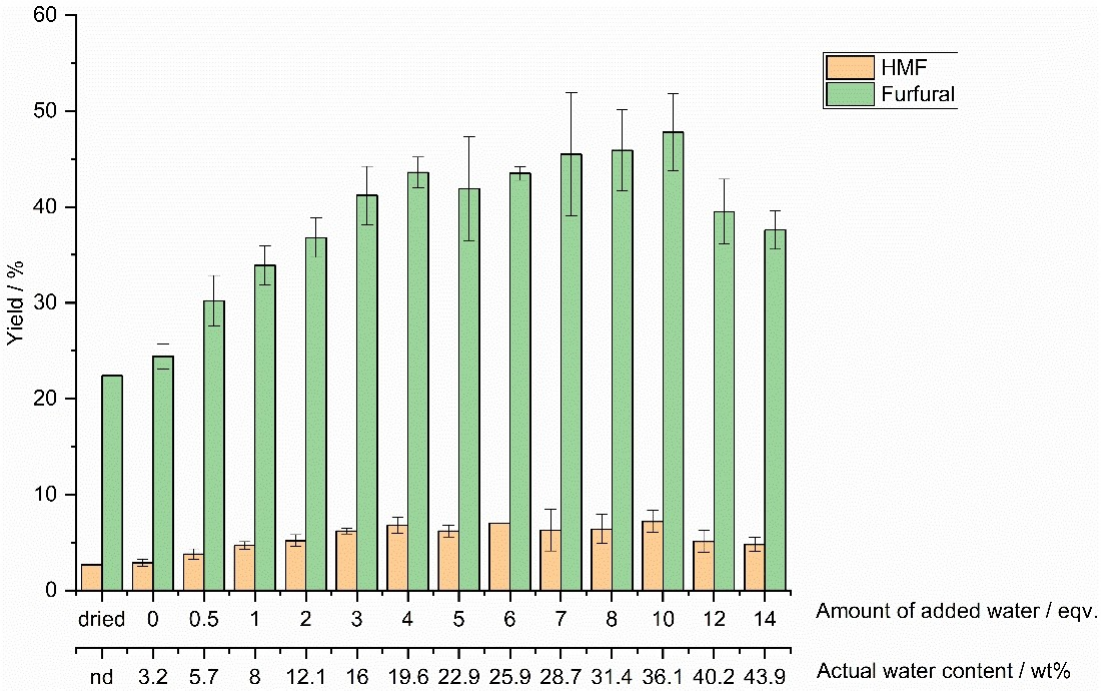
Effect of water amount in ChCl/GA/H_2_O DES on HMF and furfural yields.

Our experiments demonstrated that an increased quantity of water significantly increased the yields of furfural and HMF (Figure 3). The lowest yields for both HMF and furfural were achieved with the dried DES as the reaction medium. The furfural yield was increased from 22 % to 44 % when water addition to DES was increased from 0 to 4 equiv. (equating to the increase of actual water content from 3.2 to 19.6 wt.%). Similarly, the HMF yield increased from 3 % to 7 %. Increased yields could be connected to increased solubility of sugars in the DES or better heat and mass transfer following the decreased system viscosity.^[28]^ Increasing the added water further from 4. to 10 equiv., results were considerably varying but yields still increased. The highest yields with 10 equiv. water were determined to 48 % furfural and 7 % HMF.

### 2.3 Effect of Water Content to DES Structure

To investigate the water content that can be added to DES before the hydrogen bonds between HBD and HBA are ruptured more closely, the eutectic mixture of ChCl/GA (1 : 3) was diluted with deuterium oxide (D_2_O), and the diluted DESs were investigated by ^1^H NMR spectroscopy. A similar approach has been used to analyze the effect of water in ChCl/urea and ChCl/1,2‐propanediol DESs.[^20^, ^29^] Tests were carried out using dilutions of 16.4 %, 32.9 %, and 40.7 %, corresponding to 4, 10, and 14 equiv. of water added. Additionally, higher dilutions of 60 %, 80 %, 90 % and 95 % D_2_O solutions were made.

**Figure 4:**
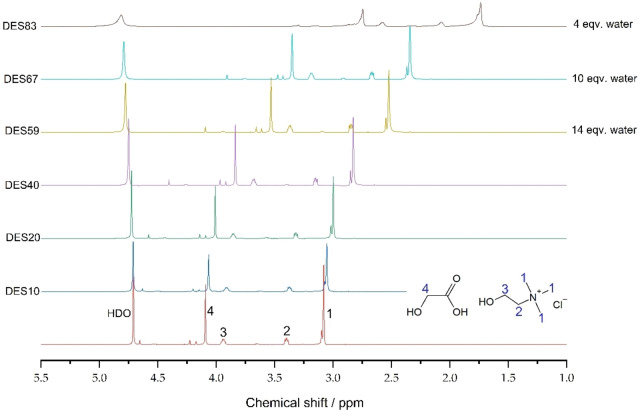
^1^H NMR spectra of D_2_O‐diluted ChCl/GA mixtures. Dilution increases from top to bottom (from 16.4 % to 95 % D_2_O).Therefore, the downfield shift of the ChCl and GA protons could indicate the increased hydration of ChCl and GA molecules.[^20^, ^29^] The downfield shift was much more significant (0.17–0.59 ppm) when the quantity of D_2_O was below 80 %, namely, in DES83, DES67, DES59, and DES40. Between DES5, DES10, and DES20 samples, signal shifts were minor (0.02–0.07 ppm), suggesting complete rupture of the hydrogen bonds between GA and ChCl.^[20]^ Therefore, this data suggested that all the water additions used in conversion reactions, namely, 14 equiv. at highest (which corresponds to the DES59 curve in Figure 4), did not rupture the complete DES hydrogen bonding structure. However, significant changes were observed below that quantity, potentially affecting the DES's properties.

The results demonstrated that, as the quantity of DES in a sample decreased, a continuous downfield shift (referred to higher energy and higher ppm) of all signals except for the peak of HDO was observed (Figure 4). It is known that the formation of C−H⋯O hydrogen bonds will enhance the deshielding effect of a proton, leading to a downfield chemical shifts of the corresponding ^1^H nuclei.[^30^, ^31^]

Interestingly, an upfield shift of the HDO signal was observed when the dilution of DES was increased (Figure 4). This is contrary to what was observed forF ChCl/urea and ChCl/1,2‐propanediol DESs.[^20^, ^29^, ^32^] However, a similar yet unexplained upfield shift was observed with ChCl/diethylene glycol DES.^[23]^ In this study authors represent that the components of DESs can facilitate hydrogen bonding among water molecules or between water and DES molecules. This increases the chemical shifts of HDO when the quantity of the components of the DES components is increased.

### 2.4 Possible HMF‐DES Interaction

In all experiments conducted so far, the yields of HMF in MIBK were considerably low at a maximum of 7 %. The next step was to try to improve the HMF yields. First, it was verified that simultaneously reacting xylose was not causing the poor yield of HMF. Thus, experiments with only glucose and xylose and the sugar mixture as feedstock were performed (Table 1, entries 1–3). Glucose produced a minor 10 % HMF yield while xylose produced a much higher furfural yield, 62 % (Table 1, entries 1–2). It is proposed that a higher reactivity of xylose compared to glucose could be connected to the less substituted C5 atom, which, according to a certain proposed mechanism, is responsible for the C2‐O attack on it (Blue path in Figure S1, Supporting Information).[^33^, ^34^, ^35^] However, this mechanism does not have any consistent observations, and the most likely pathways, according to the literature, are the aldose isomerization to ketose either through hydride shift or through enediol intermediate (Green and red paths in Figure S1, respectively).^[35]^ Isomerization is often catalyzed by Lewis acids such as ZnCl_2_.^[36]^ However, our experiments revealed that adding ZnCl_2_ as a catalyst did not improve the HMF yield, and using fructose as feedstock increased the HMF yield only from 10 % to 17 % (Table 1, entries 4–5). Additionally, since the 17 % HMF yield is very low compared to the literature (79 % yield of HMF has been reported from fructose using the ChCl/MA/H_2_O DES without additional catalyst),^[17]^ the investigation was continued.

**Table 1:** HMF and furfural yields using various feedstocks and extraction conditions.

Entry	Conditions	HMF yield [%]	Furfural yield [%]
1	Glucose as feedstock	10	–
2	Xylose as feedstock	–	62
3	Glucose/xylose as feedstock	9	49
4	ZnCl_2_ as catalyst, glucose/xylose as feedstock	10	51
5	Fructose/xylose as feedstock	17	49
6	HMF as feedstock (+additional extraction)	45 (+11)	–
7	Furfural as feedstock	–	98
8	HMF as feedstock, EtOAc extraction	50	–
9	Furfural as feedstock, EtOAc extraction	−	91
10	HMF as feedstock, optimized extraction	67	−

160 °C reaction temperature and ChCl/GA/H_2_O (1 : 3 : 6) DES were used in all reactions. When HMF/furfural was used as a feedstock, reaction time was limited to 2 minutes, otherwise, it was set at 10 minutes. MIBK was used as extracting solvent unless otherwise stated.

The next step was to place HMF into a reaction system, heat it for 2 mins at a given reaction temperature, and extract it with MIBK. As a result, it was observed that only 45 % of feed HMF was recovered from MIBK (Table 1, entry 6). When a similar setup was used for furfural, almost all furfural (98 %) was recovered from MIBK (Table 1, entry 7). Additional extraction of HMF‐containing DES increased the HMF yield to 56 %, and the HPLC analysis of the DES phase revealed that approximately 23 % of HMF was still present in the DES phase. Summing up those quantities, 21 % of HMF were not found in either the DES or the MIBK phase. When a similar test was carried out without heating the HMF/DES solution, the extracted DES contained more HMF, and only 10 % were missing. By comparing HPLC chromatograms of HMF extractions conducted with and without heating, two peaks were observed to grow when heating was used (Figure S2). Those peaks ran after the HMF peak, and their absorbances were close to that of HMF, possibly indicating that HMF‐based molecules were present in the sample. No levulinic acid was observed (either in DES or in MIBK), suggesting that HMF had not reacted further. One possible option is that HMF interacts with ChCl^[37]^ or with GA through its OH group. However, the chromatogram indicates two different molecules/interactions, one of which is also present at room temperature and the other that only forms at reaction temperature (160 °C). The interaction between HMF and DES would also explain why fructose or Lewis acids did not increase the quantity of recovered HMF. However, when the DES of the HMF reaction were analyzed with ^1^H NMR spectroscopy, there were no other signals than those from DES and those from HMF (Figure S3). Therefore, no confirmation was found for the HPCL observation, and the character of the possible interaction could not be verified using available analytical methods.

Since it was found that the HMF yield could be improved by additional extraction, the extraction procedure was optimized further. First, a reaction with HMF as feedstock was repeated, using ethyl acetate as extracting solvent. As a result, the HMF yield in the organic phase was better than using just MIBK by 5 %, leading to a 50 % yield (Table 1, entry 8). This is consistent with the HMF extraction test by Gomes et al., which revealed that EtOAc was 6 % better at extracting solvent than MIBK.^[26]^ When using ethyl acetate, 29 % of HMF was not detected either from the organic phase or from DES, which is an approximately similar order of magnitude to the missing 21 % in the MIBK reaction. Since the ethyl acetate increased the HMF yield only slightly and the furfural extraction to EtOAc was worse than to MIBK, lowering the yield from 98 to 91 % (Table 1, entry 9), MIBK was kept as an extracting solvent. However, the extraction procedure was modified. Using longer extraction time, an increased number of extractions, and a larger MIBK volume, the HMF yield increased from 45 to 67 % (Table 1, entry 10). Using an adjusted experimental setup (10 equiv. water and optimized extraction), 14 % HMF yield and 62 % furfural yield was achieved from the glucose/xylose mixture.

### 2.5 Birch Sawdust Conversion

The final step before the recycling experiments was to utilize DES with natural feedstock, birch sawdust. A mixed full factorial experimental design with central points was proposed, and the MODDE Pro software was used as a statistical tool to evaluate the reaction parameters. Factors and their levels were chosen based on previous experiments with sugars, and comprised time (10 to 30 min), temperature (150 to 170 °C), and quantity of water in DES (0.5, 4 or 10 equiv.). The solid‐to‐liquid ratio was kept constant at 0.05, and product extraction was handled as optimized in the previous section.

As shown in Table 2, the highest yield for the HMF (3.9 %) was obtained using 10 equiv. of water at 170 °C for 30 mins, while the highest furfural yield (37.5 %) was obtained using similar temperature and water quantity at a shorter reaction time of 10 mins. A shorter reaction time in furfural production compared to HMF production is common in lignocellulosic biomass since crystalline cellulose is far less reactive than amorphous hemicellulose. Therefore, the yield of HMF is usually lower compared to the yield of furfural. According to the effect plots, the quantity of water and reaction temperature are the two most important factors positively affecting both yields (Figure S4). Additionally, time had a positive effect, but was less significant. Water is required for the hydrolysis reaction of biomass to sugars, and in an earlier section it was also observed to affect sugar conversion. The ANOVA analysis (Table S1) revealed that the model used to describe the experimental data was significant.

**Table 2:** Mixed full factorial experimental design for birch sawdust conversion.

Temperature [°C]	Time [min]	Water [equiv.]	HMF yield [%]	Furfural yield [%]
150	10	0.5	0	6.2
170	10	0.5	0.4	17.3
150	30	0.5	0.3	14.2
170	30	0.5	0.5	13.1
150	10	4	0.4	10.7
170	10	4	1.7	25.9
150	30	4	1.1	21.4
170	30	4	2.2	29.2
150	10	10	0.4	7.5
170	10	10	2.9	37.5
150	30	10	1.5	26.2
170	30	10	3.9	34.6
160	20	4	0.8	23.1
160	20	4	1.3	26.8
160	20	4	1.4	24.1

There are few studies using DESs as reaction media without any additional catalyst for converting lignocellulosic biomass to HMF or furfural. Our 37.5 % furfural yield is better than the 26.4 % yield from oil palm fronds using a ChCl/OA/H_2_O DES containing 16.4 wt.% water.^[21]^ Bodachivskyi et al. reached 55 % furfural yield from softwood chips using a ChCl/OA/H_2_O DES with 7.3 wt.% water.^[38]^ Both studies used a mild reaction temperature of 100 °C but a long reaction time (135 min or 5 h, respectively), and their HMF yields were very small (1 %). Because of the modest HMF yields achieved in our study and the previous reports, DES treatment without additional catalyst could be suitable for fractionation purposes (e. g., for separating hemicellulose). Moreover, the characterization of solid residues after DES treatment (170 °C, 10 min, 10 equiv. water) revealed that no hemicellulose sugars were left in the biomass. The composition was found to be roughly half cellulose (49 %) and half lignin (52 %) (Table S2). This verified that the ChCl/GA/H_2_O DES could be suitable for sawdust fractionation before cellulose utilization. Additionally, our study demonstrated that high water loadings (32.9 wt.% of added water) could be used as part of the DES system, also when biomass is applied as feedstock.

### 2.6 Stability and Reusability of DES

**Figure 5:**
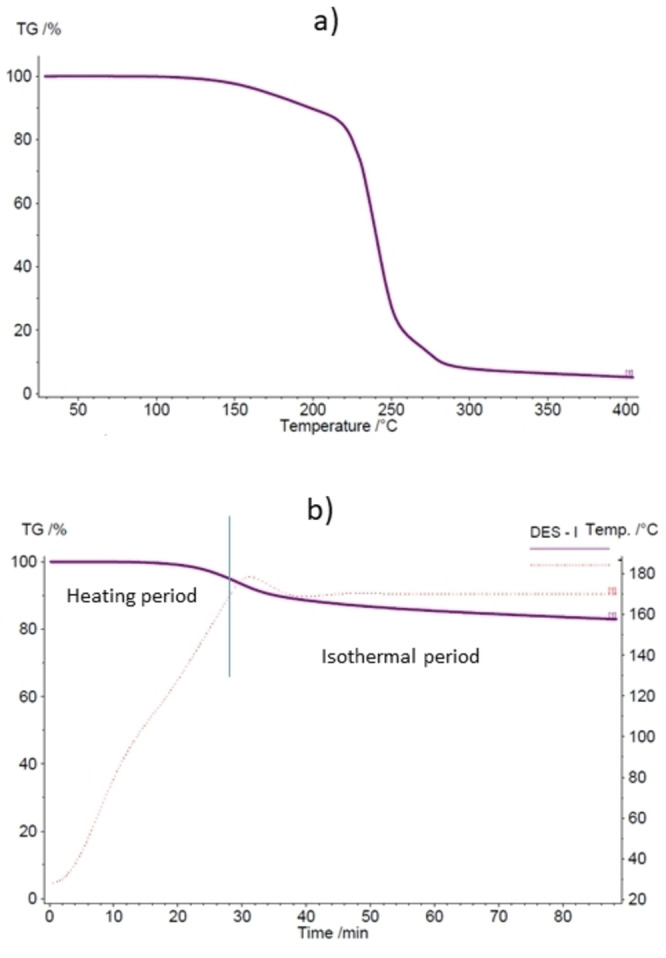
Dynamic (a) and isothermal (b) TGA curves for the ChCl/GA (1 : 3) DES.

The stability and reusability of ChCl/GA (1 : 3) DES were investigated using thermogravimetric analysis (TGA), ^1^H NMR spectroscopy, and recycling tests. Dynamic TGA revealed that the DES lost its liquid state rapidly at temperatures above 215 °C (Figure 5a). This decomposition temperature equals the value for the ChCl/GA 1 : 2 DES (218 °C) published by Rodriguez Rodriguez.^[39]^ However, a closer look at our TGA curve and that reported by Rodriguez Rodriguez reveals the decomposition to start at a temperature of 150 °C already, decreasing the mass of DES from 97.6 % to 86.6 % when the temperature rises from 150 to 215 °C. In addition to dynamic TGA, isothermal TGA was performed at the highest reaction temperature used in the conversion experiments (170 °C). It revealed that DES started to decompose already at the heating stage after 15 mins of heating. However, the decomposition was minor, decreasing the mass of DES from 100 % to 95 % during the heating period (28 min). Based on the temperature profile (dotted red line in Figure 5b), this decomposition is most likely connected to water evaporation, since a temperature of 100 °C was reached in 14 mins. The most intense mass loss happened at the beginning of the isothermal period, and after 10 mins at 170 °C, the mass of DES was decreased to 89 %. After 20 minutes at 170 °C, the mass of DES was at 87 % of the original, and after 30 mins, it had decreased to 85 %, meaning that no significant decomposition was happening anymore. After the whole isothermal period of one hour, the mass of DES had decreased to 83 %. Based on TGA experiments, DES is not fully stable at the employed reaction temperature (150–170 °C), and its decomposition depends on temperature and time. However, the experimental setup of TGA differs from that used in conversions. since a closed system and water were used and MIBK and feedstocks were present during the conversions. All these factors can affect the stability of the DES. Additionally, by using the harshest reaction conditions (170 °C, 30 min), the decomposition was determined to 15 %, which is not extreme compared to other DESs given the high temperatures.[^39^, ^40^, ^41^] Moreover, this decomposition is less than that observed for the commonly employed ChCl/OA DES (1 : 1) (completely decomposes already at 161 °C) and ChCl/lactic acid DES (1 : 2) (decomposes totally already at 140 °C).^[39]^

**Figure 6:**
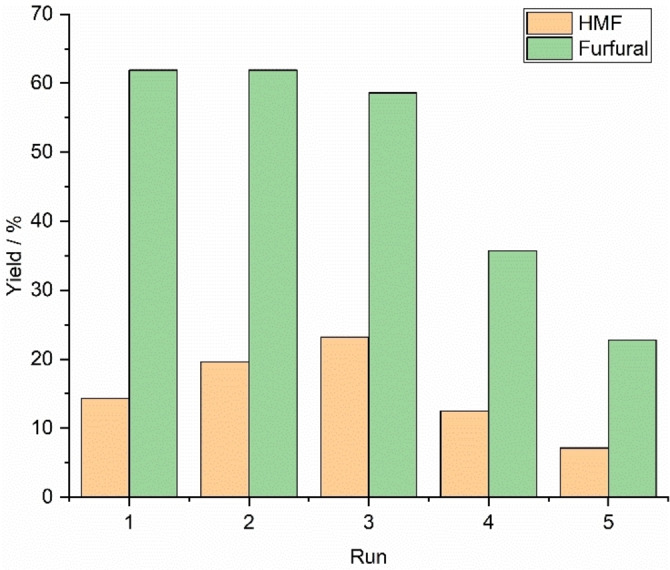
Observed HMF and furfural yields during five recycling runs.

Recycling tests were performed using the ChCl/GA/H_2_O (1 : 3 : 10) DES, sugar mix as starting material, 160 °C as reaction temperature, and 10 min as reaction time. After every cycle, the DES was filtered using cotton wool, analyzed by ^1^H NMR spectroscopy, and re‐used. Based on the yields of HMF and furfural, the DES retained its functionality for three cycles, and after that, especially the furfural yield started to decrease drastically (Figure 6). During the first two rounds, the furfural yield remained stable at 62 %, and within the third run, it decreased slightly to 59 %. In contrast, the HMF yield increased from 14 % to 23 % in the first three rounds. The increasing HMF yield was interesting, since in preliminary experiments, no glucose was left after a reaction of 10 mins at 160 °C. Again, a possible explanation may be that if HMF is interacting with the DES, interacting HMF would be released from DES as more HMF forms. However, after three rounds, both yields started to decrease, and the appearance of the DES changed to very viscose. In the ^1^H NMR spectra of the DESs, no HMF or furfural were visible after recycling runs 1 and 2 (Figure S11). Instead, characteristic peaks for MIBK were observed after the first run, and they were present after that in each recycled sample (Figure S11), meaning that part of the MIBK was dissolved in the DES. After the last three runs, HMF was also observed in the DES in the corresponding ^1^H NMR spectra and its quantity appeared to increase with every cycle (Figure S11).

## 3 Conclusion

This study focused on the conversion of C5/C6 sugar mixtures to valuable chemicals. Glucose/xylose mixtures were converted to furfural and HMF in the ChCl/GA/H_2_O DES/MIBK system without any additional catalyst. Additionally, the conversion of birch sawdust was carried out. Results demonstrated that the presence of water in DESs enhances the formation of furfural and HMF for both starting materials. Based on ^1^H NMR spectra, it was concluded that 10 equiv. of water (32.9 wt.%) did not rupture the DES hydrogen bonding structure but were beneficial for conversion. The formation of furfural was successful, resulting in a 62 % yield from the sugar mixture and 37.5 % yield from birch sawdust. The formation of HMF suffered from poor yields, which, for sawdust, was however found beneficial since the treatments appeared to fractionate the sawdust hemicellulose to furfural while most cellulose and lignin remained solid. Low yields of HMF from sugars were connected to possible HMF‐DES interaction. Finally, the recyclability of the DES was studied, and it was observed that the DES could be recycled for three runs. Following this, furfural yields started to decrease significantly, and the appearance of DES changed. Overall, the present work introduces novel information on high water content DESs and introduces a green process for simultaneous biomass fractionation and furfural production.

## Experimental Section

### Materials

All chemicals (choline chloride (ChCl) >98 %, glycolic acid (GA) 98 %, methyl isobutyl ketone (MIBK) 99.5 %, glucose >99 %, xylose >99 %, fructose >99 %, ZnCl_2_ ≥95 %, ethyl acetate (EtOAc) 99.7 %, furfural 98 %, HMF 98 %, dimethyl sulfone (*Trace*CERT), and deuterium oxide (D_2_O) 99.96 % were used as received without further purification. ChCl and GA were stored in a desiccator to keep their water content constant. Birch sawdust (*Betula pendula*) was received from a sawmill in Northern Sweden, and its chemical composition is presented in Table S2. Sawdust was dried to constant weight and stored in an oven (50 °C) before use.

### Conversion Procedure

For each conversion reaction, the DES was individually prepared before conversion. ChCl (6.8 mmol) and GA (20.4 mmol) were weighed into a microwave reaction tube. The tube was closed, heated, and stirred vigorously in a 70 °C oil bath until a colorless liquid was obtained. After complete ChCl/GA DES formation, water was added into the solution (0‐14 equiv. with respect to ChCl). Water‐containing DES is labelled as ChCl/GA/H_2_O.

For the conversion of monosaccharide mixtures, glucose (0.44 mmol, 80 mg), xylose (0.32 mmol, 48 mg), and MIBK (2 mL) were added to ChCl/GA/H_2_O DES. The closed reaction tube was heated in a Biotage Initiator microwave reactor at 150–170 °C, using a 10–30 min reaction time. After the reaction, the tube was cooled, and the MIBK layer was separated using a syringe and needle. DES was further extracted with 2×2 mL MIBK or after the extraction optimization with 3×6 mL MIBK. Extracted MIBK solutions were combined and analyzed with HPLC to determine HMF and furfural. Before the HPLC analysis, samples were diluted with methanol (200 μL sample, 800 μL methanol) to prevent peak tailing and filtered with 0.45 μm syringe filters. All conversions were performed at least as duplicates.

For the conversion of birch sawdust, 128/150/186 mg sawdust was used as starting material. The quantity of sawdust varied according to the water (63/490/1225 μl) in DES, since the solid to liquid ratio was kept constant at 0.05. Otherwise, the reaction procedure was similar to the monosaccharide mixture detailed above. After the reaction, DES was extracted with 3×6 mL MIBK, and the extracted solution was evaporated into a smaller solvent volume. The concentrated MIBK solution was diluted with methanol and filtered with a 0.45 μm syringe filter prior to the HPLC analysis.

Recycling experiments were performed using glucose (0.44 mmol, 80 mg) and xylose (0.32 mmol, 48 mg) as starting materials, MIBK (2 mL) as the organic phase, and reaction conditions of 160 °C and 10 min. After every run, the organic phase was separated, and DES was extracted with 3×6 mL MIBK. After extraction, DES was filtered through cotton wool and used again with a new load of glucose, xylose, and MIBK. Recycling was repeated for five runs.

### Product Analysis

HMF and furfural were analyzed with Waters 2695 separation module and Waters 996 photodiode array (PDA) detector. An Atlantis T3 (3 μm, 4.6×150 mm) or Atlantis dC18 column (5×μm, 4.6×150 mm) was used when HMF and furfural were determined either from the organic phase or from DES, respectively. A mixture of water (0.1 % TFA) and methanol (0.1 % TFA) (90 : 10) was used as the mobile phase, with a flow rate of 1 mL min^−1^. The column temperature was kept constant at 30 °C. UV detection for HMF was performed at 284 nm, while for furfural, 277 nm was used as wavelength. Calibrations were performed using commercial HMF and furfural, and all samples were analyzed as duplicates. Yields are expressed as the ratio of furfural or HMF obtained in the organic phase to the initial xylose or glucose fed to the reaction.

### Characterization of Sawdust Before and After DES Treatment

Characterization of native sawdust was carried out similar to that described in a previous study.^[40]^ Additionally, lignin, cellulose, and hemicellulose contents of solid residue produced by DES treatment were analyzed. Determination of lignin was carried out using a similar procedure as with native sawdust, according to Sluiter et al.^[43]^ Cellulose and hemicellulose contents were determined by utilizing total hydrolysis of residue and then measuring the glucose and xylose contents of hydrolysate via HPLC.^[43]^ HPLC detection of glucose and xylose was carried out using Shimadzu LC‐20AT liquid chromatograph instrument fitted with a SIL‐20A TH autosampler, RID‐20A refractive index detector, SUGAR SH‐G pre‐column, and Shodex SUGAR SH1821 column (8.0×300 mm). Sulfuric acid (5 mM) was used as a mobile phase with a flow rate of 0.8 mL min^−1^, and the column temperature was kept constant at 60 °C.

### ^1^H NMR Spectroscopy

^1^H NMR spectroscopy was used to determine the water content of ChCl/GA DES, investigate the effect of water dilution on the DES structure, and monitor the changes in DES during multiple reaction runs. All spectra were recorded with a Bruker Ascend 400 MHz spectrometer using different parameters detailed below.

To determine the water content of ChCl/GA DES, water contents of both starting materials of DES (ChCl and GA) were analyzed with NMR spectroscpy. ^1^H NMR spectra were recorded using dimethyl sulfone as internal standard and D_2_O as solvent. Analysis was carried out at room temperature, using 64 scans, 4 dummy scans, 60 s relaxation delay (D1), and 4 s acquisition time. The water content of D_2_O was determined using a control sample, and it was subtracted from the real samples. After calculating the water contents of both starting materials (Supplementary Information), these values were used to calculate the water content of DES, based on the masses ChCl and GA in DES.

To investigate the effect of water dilution on DES's structure, ^1^H NMR spectra of D_2_O‐diluted DESs were recorded at room temperature with 16 scans and 1 s relaxation delay (D1). A ChCl sample in D_2_O was measured as a control. Additionally, changes at DES during recycling were monitored by measuring ^1^H NMR spectra before the first reaction and after every recycling cycle. Samples were prepared in D_2_O and measured using similar parameters described above.

### TGA

TGA was carried out to study the stability of ChCl:GA DES in reaction conditions. Two different analyses were carried out using Netzsch STA449F3 thermogravimetric analyzer: isothermal analysis (1 h at 170 °C) and dynamic analysis with increasing temperature (5 °C min^−1^ from 28 to 405 °C). Accurately weighed samples (≈200 mg) were placed into Al_2_O_3_ crucibles and heated to the desired temperature under nitrogen flow (250 mL min^−1^).

## Conflict of interest

The authors declare no conflict of interest.

## Supporting information

As a service to our authors and readers, this journal provides supporting information supplied by the authors. Such materials are peer reviewed and may be re‐organized for online delivery, but are not copy‐edited or typeset. Technical support issues arising from supporting information (other than missing files) should be addressed to the authors.

Supporting Information
