# Supplementary material for: Furfural and 5‐Hydroxymethylfurfural Production from Sugar Mixture Using Deep Eutectic Solvent/MIBK System
Source: ChemistryOpen. 2021 Oct 7;10(10):1004–12. doi: 10.1002/open.202100163 (PMC8495682; doi:10.1002/open.202100163)
Supplement: Supplementary file 1 — Supporting Information [file OPEN-10-1004-s001.pdf]

ChemistryOpen

Supporting Information

Furfural and 5-Hydroxymethylfurfural Production from Sugar Mixture Using Deep Eutectic Solvent/MIBK System

Annu Rusanen, Katja Lappalainen, Johanna Kärkkäinen, and Ulla Lassi*

Author Contributions

A.R. Conceptualization:Lead; Data curation:Lead; Funding acquisition:Equal; Investigation:Lead; Methodology:-Lead; Validation:Lead; Writing – original draft:Lead; Writing – review & editing:Lead
K.L. Conceptualization:Supporting; Data curation:Equal; Funding acquisition:Equal; Investigation:Supporting; Methodology:Supporting; Supervision:Equal; Writing – original draft:Equal; Writing – review & editing:Equal
J.K. Conceptualization:Supporting; Data curation:Supporting; Investigation:Supporting; Methodology:Supporting; Supervision:Equal; Writing – original draft:Equal; Writing – review & editing:Equal
U.L. Conceptualization:Supporting; Data curation:Supporting; Funding acquisition:Equal; Investigation:Supporting; Project administration:Lead; Supervision:Equal; Writing – original draft:Equal; Writing – review & editing:Equal

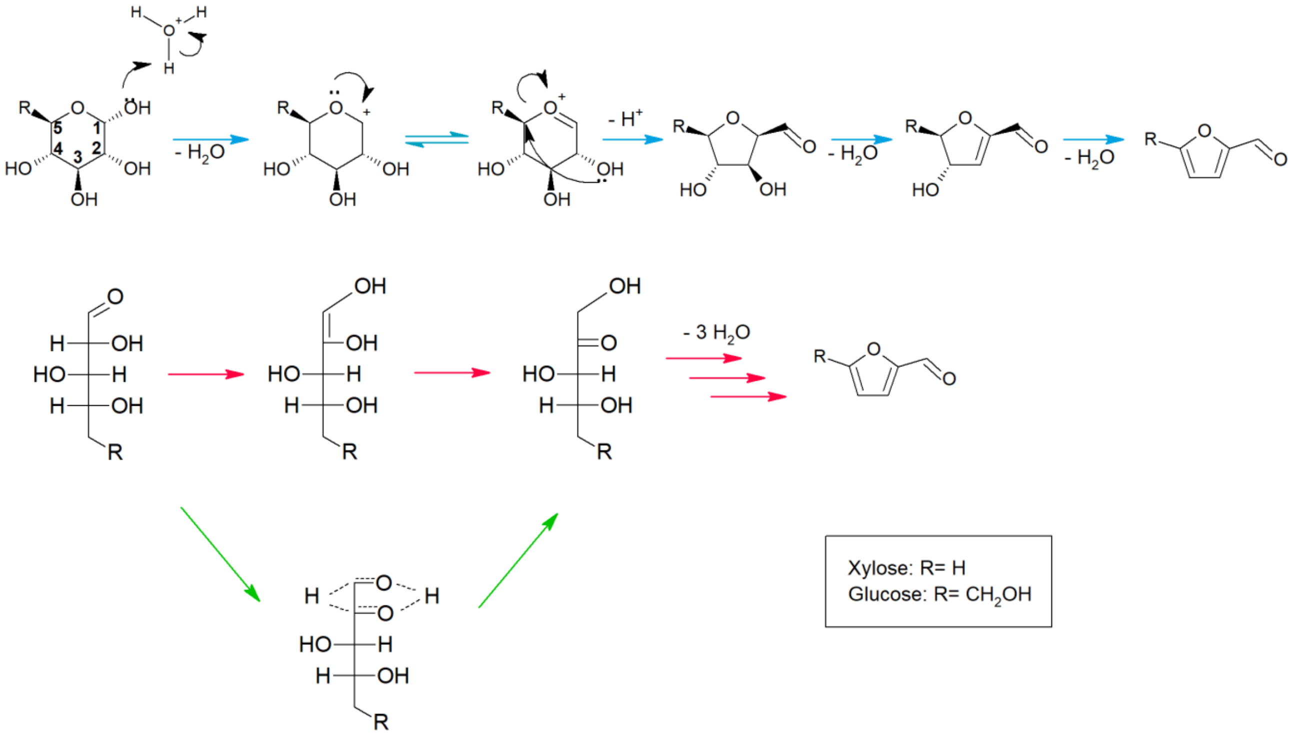

Fig. S1. Proposed pathways for aldose dehydration to furan derivatives by: protonation of C1 hydroxyl and ring contraction after C2O attack on C5 (blue), aldose isomerization to ketose through hydride shift (red) and aldose isomerization to ketose through enediol intermediate (green). Reference: Istasse, T., & Richel, A. (2020). Mechanistic aspects of saccharide dehydration to furan derivatives for reaction media design. *RSC Advances*, *10*(40), 23720-23742.

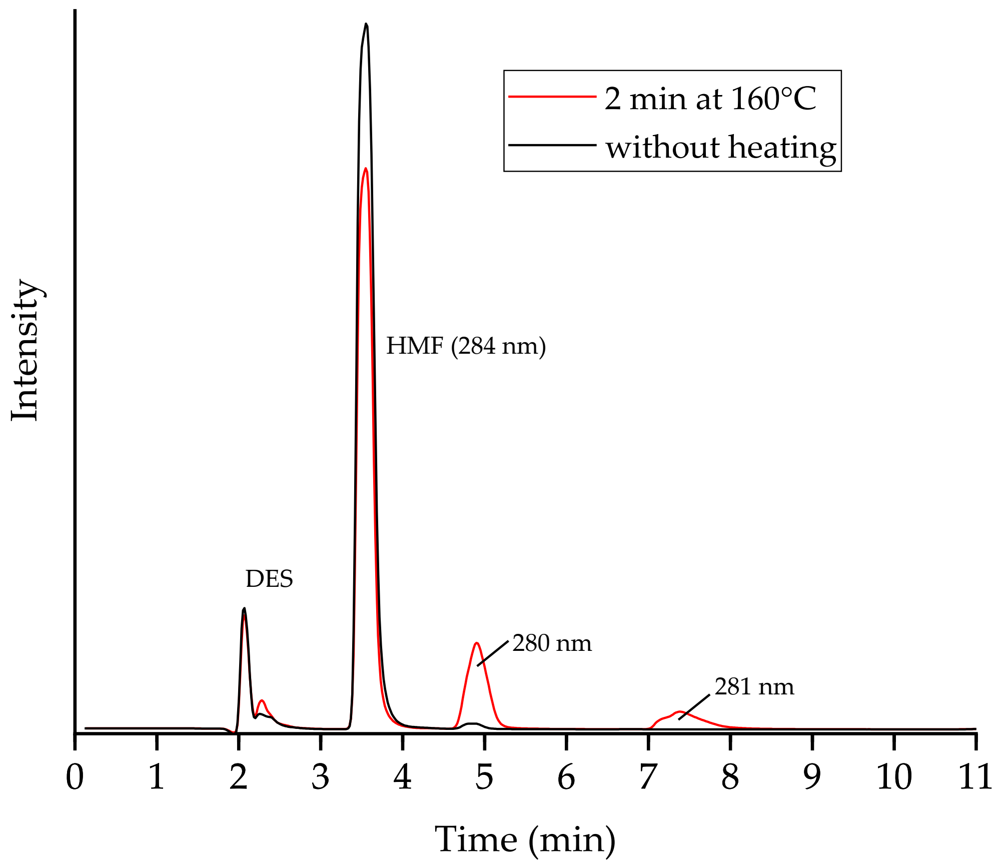

Fig. S2. HPLC chromatogram of DES phase when HMF was used as starting material

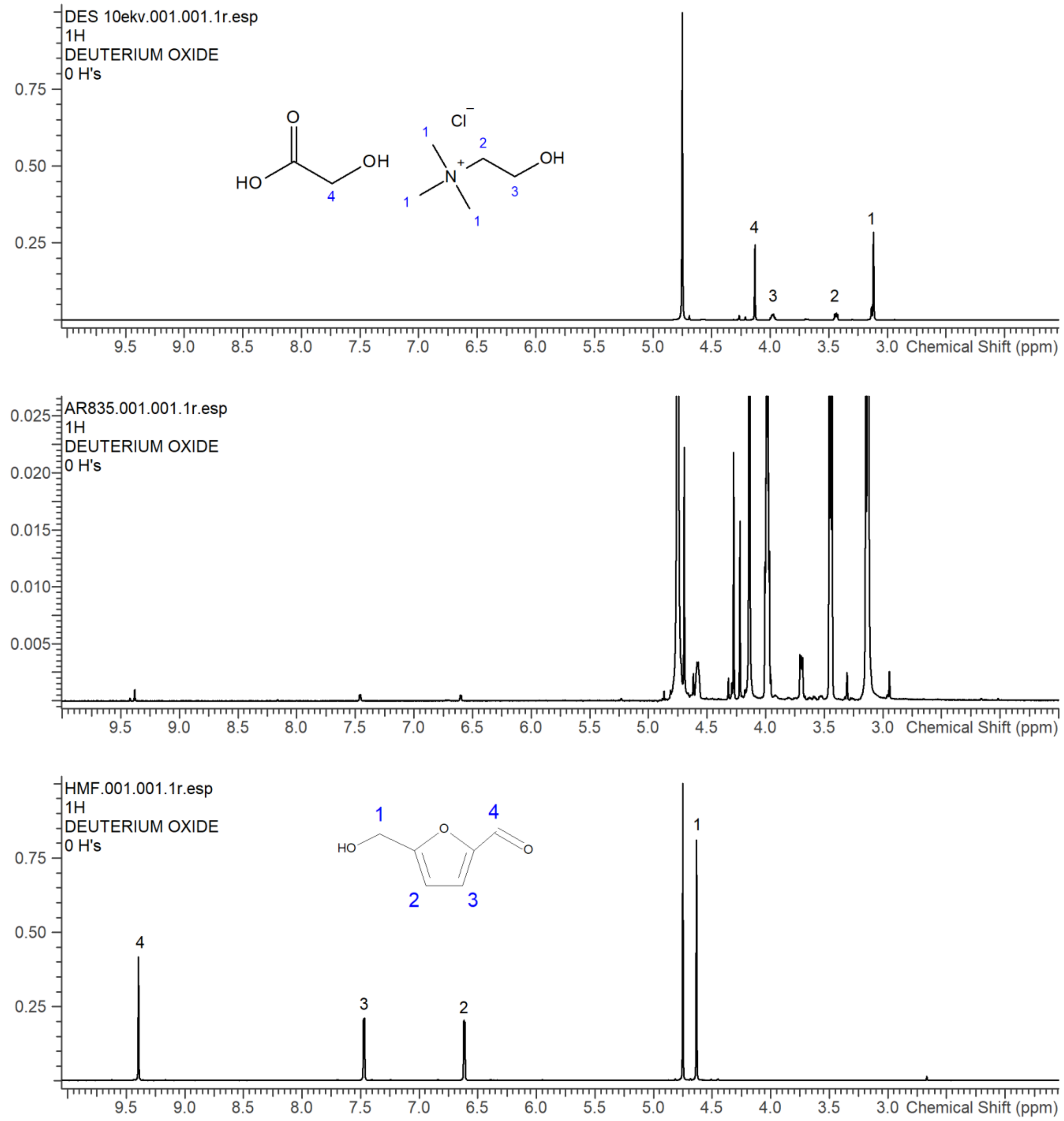

Fig. S3. NMR spectrum of DES after HMF reaction (middle) shows no other peaks than those from unused DES and from HMF.

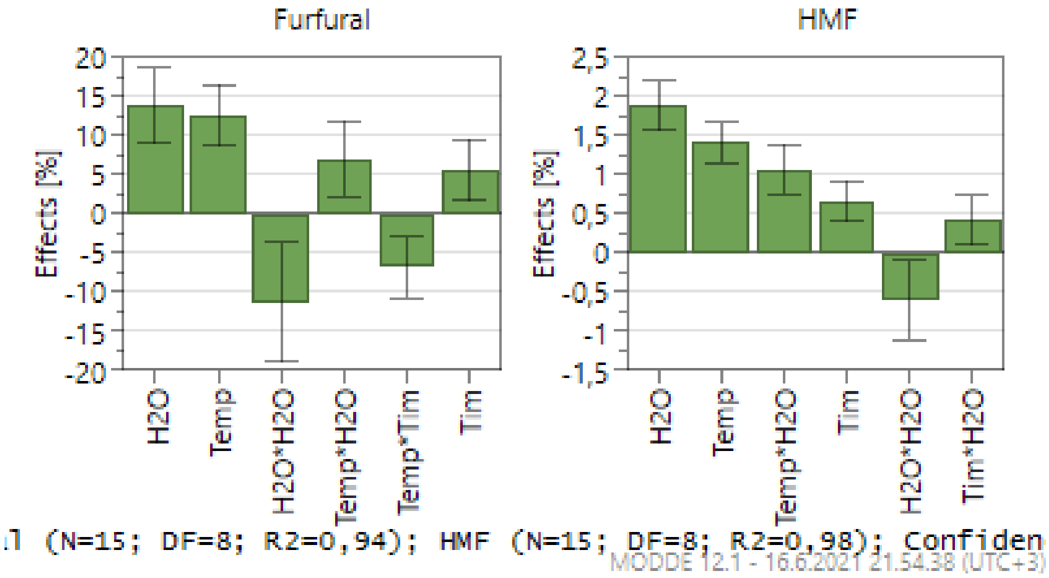

Fig S4. Effect plot of sawdust conversion to furfural and HMF.

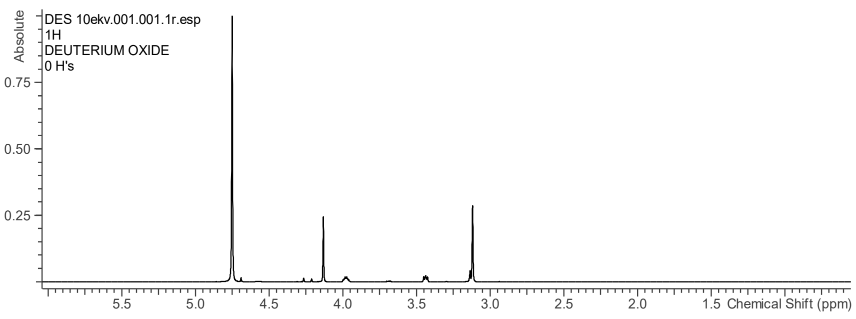

Figure S5. NMR spectrum of unused DES.

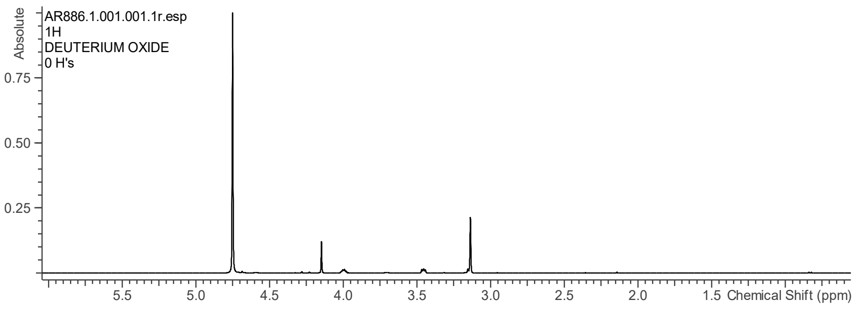

Figure S6. NMR spectrum of DES after first round.

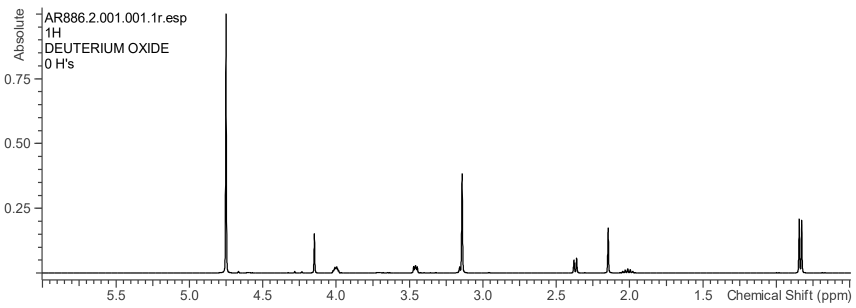

Figure S7. NMR spectrum of DES after second round.

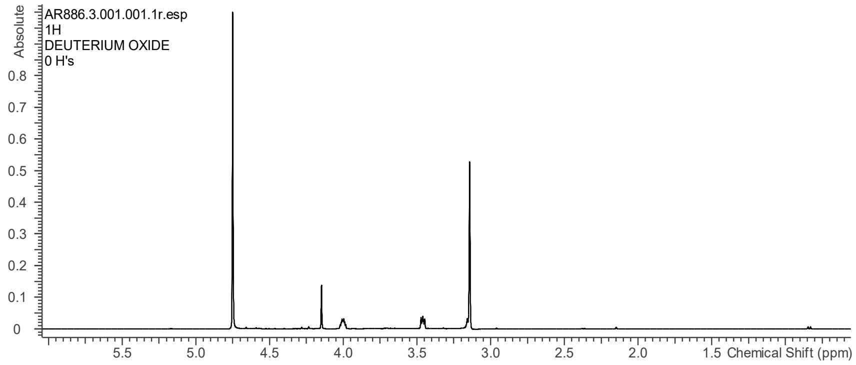

Figure S8. NMR spectrum of DES after third round.

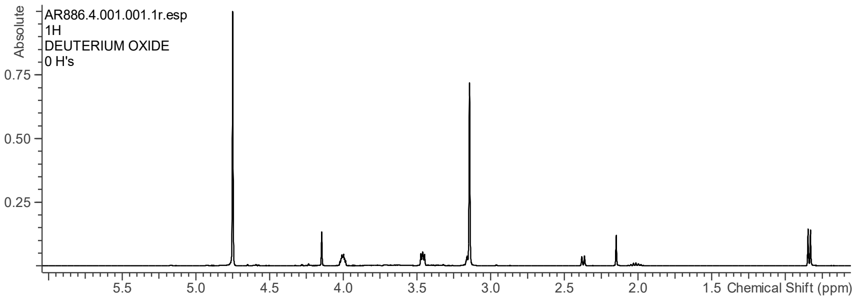

Figure S9. NMR spectrum of DES after fourth round.

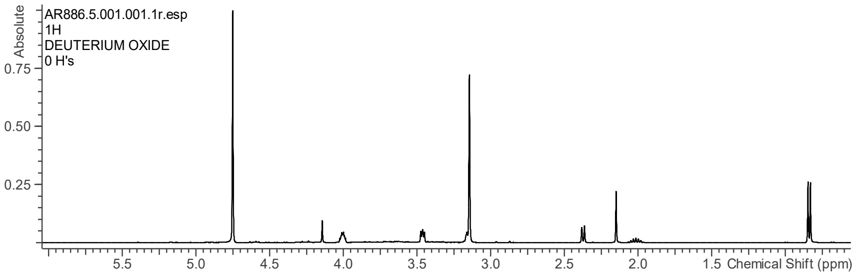

Figure S10. NMR spectrum of DES after fifth round.

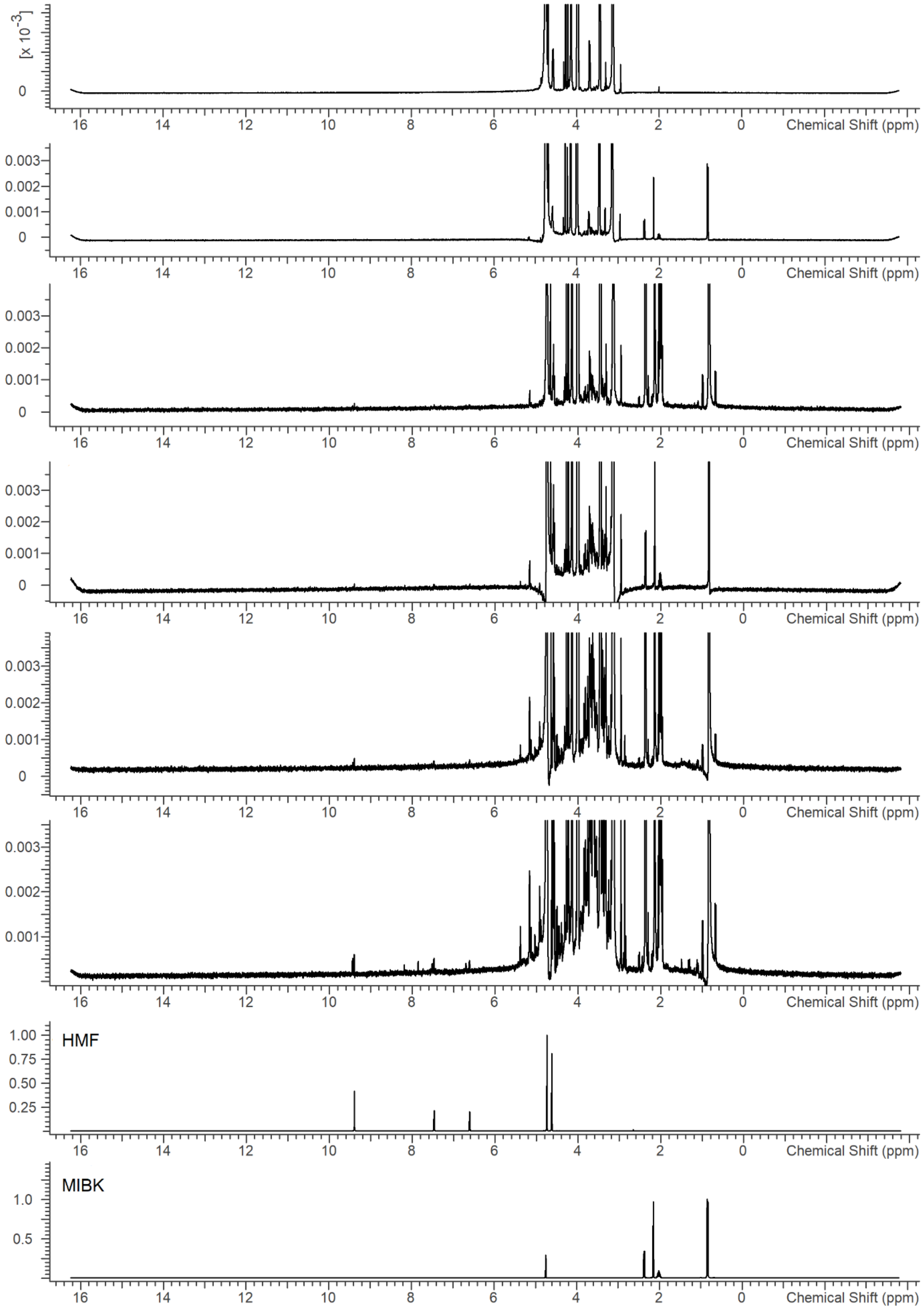

Figure S11. Zoomed ^{1}H NMR spectra (originals presented in Fig.S5-S10) from top to bottom: unused, after first round, after second round, after, third round, after fourth round and after fifth round. Also HMF and MIBK spectra are presented.

Table S1. ANOVA analysis

**Furfural**	**DF**	**SS**	**MS (variance)**	**F**	**p**	**SD**
Total	15	7985,64	532,376			
Constant	1	6733,12	6733,12			
Total corrected	14	1252,52	89,4655			9,45862
Regression	6	1183,03	197,171	22,6994	**0,000**	14,0418
Residual	8	69,4894	8,68617			2,94723
Lack of Fit	6	62,1627	10,3605	2,82815	**0,284**	3,21877
(Model error)						
Pure error	2	7,32666	3,66333			1,91398
(Replicate error)						
	N = 15	Q2 =	0,737	Cond. no. =	3,077	
	DF = 8	R2 =	0,945	RSD =	2,947	
		R2 adj. =	0,903			

**HMF**	**DF**	**SS**	**MS (variance)**	**F**	**p**	**SD**
Total	15	39,92	2,66133			
Constant	1	23,5627	23,5627			
Total corrected	14	16,3573	1,16838			1,08092
Regression	6	16,058	2,67634	71,5394	**0,000**	1,63595
Residual	8	0,299286	0,0374107			0,193419
Lack of Fit	6	0,0926191	0,0154365	0,149386	**0,970**	0,124244
(Model error)						
Pure error	2	0,206667	0,103333			0,321455
(Replicate error)						
	N = 15	Q2 =	0,966	Cond. no. =	3,077	
	DF = 8	R2 =	0,982	RSD =	0,1934	
		R2 adj. =	0,968			

Table S2. Chemical characterization of native birch sawdust and residue of it after DES treatment. Results are presented as % of dry matter content.

	Native birch sawdust (%)	Residue after DES treatment (%)
**Ash**	1	ND
**Extractives**	4	ND
**Lignin**	24	52
Holocellulose	71	ND
**Cellulose***	*38*	49[a]
**Hemicellulose**	*33*	0[b]
• **Xylose**	***67*	ND
• **Glucose**	***10*	ND
• **Mannose**	***4*	ND
• **Galactose**	***4*	ND
• **Arabinose**	***3*	ND
• **Rhamnose**	***2*	ND

* α-Cellulose ** from hemicellulose

[a] determined after total hydrolysis as glucose

[b] determined after total hydrolysis as xylose

ND=not determined

Determination of water content of ChCl and GA was performed using formula:

$$Amount\ of\ water\ (wt\%) = \frac{n_{IC} \times Int_t \times M_t \times m_{IC}}{n_t \times Int_{IC} \times M_{IC} \times m_s} \times 100\%$$

where n is number of protons giving rise to NMR signal, M is molar mass (g/mol), m is mass (mg) and Int is integral of NMR signal. IC refers to internal calibrant (dimethyl sulfone), t to target molecule (ChCl or GA) and s to sample.
